# Prevention of Gastrointestinal Bleeding in Patients Receiving Direct Oral Anticoagulants: A Narrative Review and Practical Framework for Prescribers

**DOI:** 10.3390/clinpract16070120

**Published:** 2026-06-26

**Authors:** Nicoleta Dubei, Larisa Anghel, Laura-Cătălina Benchea, Radu Andy Sascău, Cristina Prisacariu, Mircea Ovanez Balasanian, Bogdan-Sorin Tudurachi, Bianca-Ștefania Profire, Cristian Stătescu

**Affiliations:** 1 Internal Medicine Department, “Grigore T. Popa” University of Medicine and Pharmacy, 700503 Iași, Romania; nicoletadubei@yahoo.com (N.D.); benchea.lauracatalina@yahoo.com (L.-C.B.); radu.sascau@umfiasi.ro (R.A.S.); cprisacariu88@gmail.com (C.P.); ovanes718@yahoo.com (M.O.B.); bogdan-sorin.tudurachi@d.umfiasi.ro (B.-S.T.); bianca-stefania.profire@umfiasi.ro (B.-Ș.P.); cristian.statescu@umfiasi.ro (C.S.); 2 Cardiology Department, Cardiovascular Diseases Institute “Prof. Dr. George I. M. Georgescu”, Iași 700503, Romania

**Keywords:** DOAC, gastrointestinal bleeding, risk factors, prevention, *Helicobacter pylori*, NSAIDs

## Abstract

**Background/Objectives**: As population aging increases the prevalence of atrial fibrillation (AF), the use of direct oral anticoagulants (DOACs) has expanded for thromboembolism prevention. Although DOACs offer advantages over vitamin K antagonists (VKAs), gastrointestinal bleeding (GIB) remains the most common extracranial adverse event. Current guidelines address global bleeding risk but provide limited guidance on site-specific gastrointestinal risk assessment and prevention. This narrative review aims to summarize current evidence on the mechanisms, etiologies, and risk factors for DOAC-associated gastrointestinal bleeding and to propose a pragmatic, risk-based framework to support clinicians in individualized bleeding prevention. **Methods**: A narrative review of studies published between 2004 and 2025 was conducted, including randomized clinical trials, real-world evidence, meta-analyses, and major society guidelines. Evidence addressing DOAC safety profiles, gastrointestinal bleeding etiologies, patient-level risk factors, medication interactions, and preventive strategies was analyzed. **Results**: Gastrointestinal bleeding in patients treated with DOAC is strongly influenced by underlying gastrointestinal pathology, comorbid conditions, and concomitant medications. Established risk factors include prior gastrointestinal hemorrhage, *Helicobacter pylori* infection, gastrointestinal malignancy, diverticulosis, and angiodysplasia, as well as the use of nonsteroidal anti-inflammatory drugs (NSAIDs), antiplatelet therapy, or selective serotonin reuptake inhibitors (SSRIs). DOACs differ in gastrointestinal safety: apixaban consistently demonstrates the most favorable profile, whereas rivaroxaban and high-dose dabigatran show higher GIB rates. Preventive strategies such as *H. pylori* testing and eradication, proton pump inhibitor use in high-risk individuals, avoidance of NSAIDs and unnecessary antiplatelet therapy, and individualized DOAC selection may help reduce bleeding risk. **Conclusions**: Gastrointestinal bleeding risk in patients receiving DOAC therapy should be assessed using a site-specific and dynamic approach. A structured strategy integrating baseline risk evaluation, correction of modifiable factors, tailored anticoagulant selection, and risk-adapted follow-up may improve the safety of anticoagulation. The proposed framework may provide a pragmatic approach to individualized bleeding risk mitigation while preserving the benefits of DOAC therapy; however, prospective validation is required before its routine implementation can be recommended.

## 1. Introduction

Atrial fibrillation (AF) is projected to increase substantially over the coming decades, primarily due to population aging [1]. In parallel, the clinical use of direct oral anticoagulants (DOACs) has expanded. Large randomized trials—ARISTOTLE, RE-LY, ROCKET-AF, and ENGAGE AF–TIMI 48—have shown that DOACs are superior or non-inferior to warfarin for the prevention of ischemic stroke and systemic embolism, while consistently reducing intracranial hemorrhage and all-cause mortality [2,3,4,5,6].

From a practical standpoint, DOACs offer predictable pharmacokinetics, rapid onset of action, and minimal need for routine laboratory monitoring [7]. Despite these advantages, bleeding remains a clinically relevant complication, with gastrointestinal bleeding (GIB) representing a major proportion of events. Approximately 40% of DOAC-associated major bleeding episodes occur within the gastrointestinal tract [8,9,10]. Accordingly, a clear understanding of mechanisms, predisposing conditions, and modifiable contributors to GIB is essential to maximize the net clinical benefit of anticoagulation.

The selection of the appropriate DOAC and dose carries important clinical implications. Current guidelines recommend dose reduction only when prespecified criteria are met; dose reduction outside these criteria (i.e., “off-label” underdosing) may reduce efficacy without reliably improving safety. Beyond immediate clinical management, the prognostic impact of GIB is considerable. Thirty-day mortality after major gastrointestinal hemorrhage has been estimated at 8.4% [11], and one-year mortality is higher among patients who experience GIB than among those who do not [12]. In a case–control analysis, Verso et al. reported that DOAC-treated patients with GIB had a markedly increased risk of one-year mortality (adjusted OR 7.04; 95% CI 3.82–14.31) compared with patients without bleeding [12].

Beyond its prognostic impact, gastrointestinal bleeding in anticoagulated patients also carries a substantial economic burden. In a large population-based time-series study of over 59,000 hospitalizations for gastrointestinal bleeding among anticoagulated patients with atrial fibrillation in Ontario, Canada (2003–2021), the widespread uptake of DOACs after 2012 was not associated with a significant reduction in GI bleeding-related hospitalization costs [13]. Although DOAC adoption led to significant declines in intracranial hemorrhage rates and costs, gastrointestinal bleeding rates initially increased following the transition from vitamin K antagonists, before declining gradually over time. These findings illustrate that the shift to DOACs—despite overall improved safety—has not translated into reduced costs for gastrointestinal bleeding, reinforcing the clinical and economic rationale for the preventive strategies outlined in this review.

The 2024 European Society of Cardiology (ESC) Guidelines emphasize that bleeding-risk scores (e.g., HAS-BLED) should not be used to withhold or discontinue anticoagulation in patients who otherwise meet indications, but rather to identify modifiable risk factors and to guide their correction [14]. However, these recommendations primarily address global bleeding risk and do not provide an exhaustive, site-specific framework for the prevention of gastrointestinal bleeding. Importantly, bleeding risk is not uniform across anatomical sites. The determinants of intracranial hemorrhage differ from those driving gastrointestinal bleeding, which is often closely linked to underlying digestive pathology and modifiable clinical factors. Despite this, current approaches to bleeding risk assessment remain insufficiently tailored to gastrointestinal-specific mechanism and clinical scenarios encountered in routine practice.

Because GIB is the most common major bleeding site among patients receiving DOACs, a systematic approach to evaluating gastrointestinal, patient-related, and treatment-related risk factors is warranted. These determinants may vary across countries, reflecting population characteristics and differences in healthcare delivery. In settings with limited access to screening programs for digestive neoplasia, delayed diagnosis may contribute to preventable bleeding events [12].

At present, clinicians lack practical tools to integrate gastrointestinal risk factors, comorbidities, and treatment-related variables into individualized decision-making. This gap is particularly relevant given the high prevalence of occult gastrointestinal lesions and the potential for anticoagulation to unmask underlying pathology.

Existing reviews focus mainly on comparative bleeding risk and post-bleeding management; however, a structured, site-specific prevention framework before and during DOAC therapy remains insufficiently developed.

The present review is specifically directed at prescribing clinicians—including internists, cardiologists, and general practitioners—and focuses on strategies to prevent gastrointestinal bleeding before and during DOAC therapy. The management of active gastrointestinal hemorrhage is beyond its scope and is addressed in dedicated gastroenterology guidelines.

Although most of the cited evidence derives from patients with atrial fibrillation—reflecting the predominance of this indication in the available evidence—the prevention framework proposed in this manuscript is intended to apply broadly to all patients receiving DOAC therapy. Gastrointestinal bleeding risk assessment and risk factor modification are warranted at DOAC initiation regardless of the underlying indication, as gastrointestinal risk is primarily determined by patient-specific factors. Clinicians should additionally consider indication-specific factors, including thromboembolic risk, anticipated duration of anticoagulation, and the clinical consequences of treatment interruption, when individualizing prevention strategies.

In this context, this review aims to move beyond a descriptive synthesis of the literature by integrating current evidence into a pragmatic, risk-based clinical framework for the prevention of DOAC-associated gastrointestinal bleeding. In addition, we highlight key areas of clinical uncertainty and discuss how these may influence individualized management strategies in real-world clinical practice.

## 2. Methods

This narrative review was conducted to summarize and synthesize contemporary evidence on the mechanisms, etiologies, risk factors, and preventive strategies related to gastrointestinal bleeding in patients treated with direct oral anticoagulants. A structured, but non-systematic, literature search was performed across PubMed/MEDLINE, Scopus, Web of Science, and Embase for studies published between January 2004 and August 2025. The search strategy included the following terms: “direct oral anticoagulants”, “DOAC”, “gastrointestinal bleeding”, “atrial fibrillation”, “bleeding risk”, “*Helicobacter pylori*”, “NSAIDs”, “antiplatelet therapy”, “selective serotonin reuptake inhibitors (SSRIs)”, “upper gastrointestinal bleeding”, and “lower gastrointestinal bleeding”.

Eligible sources included randomized controlled trials, meta-analyses, systematic reviews, real-world observational studies, and clinical practice guidelines addressing any of the following: (1) comparative gastrointestinal safety of DOACs; (2) mechanisms or etiologies of GIB; (3) clinical or medication-related risk factors; and (4) strategies for GIB prevention in anticoagulated patients. Case reports, small case series, and studies unrelated to gastrointestinal outcomes were excluded.

**Figure 1:**
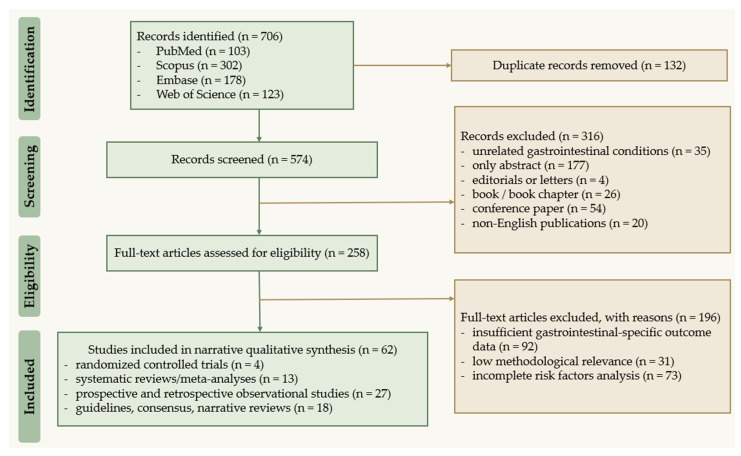
Preferred Reporting Items for Systematic reviews and Meta-Analysis (PRISMA) diagram.

Two reviewers (N.D. and L.A.) independently screened titles and abstracts, with full-text evaluation of relevant articles. Disagreements were resolved by consensus with a third reviewer (C.S.). Data were synthesized qualitatively, given the heterogeneity of study designs and reported outcomes. This review was not designed as a systematic review and does not aim to provide a formal quantitative synthesis of evidence, but rather to offer a clinically oriented interpretation of the available literature. The article selection process is summarized in Figure 1.

## 3. Comparative Risk of Gastrointestinal Bleeding with DOACs

### 3.1. Evidence from Randomized Trials

Compared with vitamin K antagonists (VKAs), DOACs are preferred in contemporary practice due to a more favorable efficacy–safety balance, including reductions in stroke, intracranial hemorrhage, and all-cause mortality [6]. Additional advantages derive from their pharmacokinetic profile—rapid onset of action, predictable exposure, no requirement for routine coagulation monitoring, and fewer clinically relevant drug–drug and drug–food interactions [7].

Despite these benefits, bleeding risk remains a major limitation, with gastrointestinal bleeding (GIB) representing a prominent concern. In a meta-analysis published in Gastroenterology, Holster et al. (2013) pooled 17 randomized clinical trials comprising more than 75,000 patients and reported an overall increase in GIB with DOACs (apixaban, dabigatran, rivaroxaban, edoxaban) compared with warfarin, heparin, or placebo (RR 1.45; 95% CI 1.07–1.97) [15]. In the same analysis, extracranial bleeding—particularly GIB—was higher with DOACs than with warfarin, with clinically meaningful variability according to molecule and dose [15]. Across studies, approximately 40% of DOAC-associated major bleeding events occur in the gastrointestinal tract [8,9,10].

Data from pivotal randomized trials further suggest important intra-class differences in gastrointestinal safety. Apixaban generally demonstrates the most favorable GI profile, with rates of GIB comparable to or modestly lower than those observed with warfarin and signals toward fewer severe bleeding events and lower post-event mortality. Dabigatran shows a dose-dependent pattern: the 220 mg/day regimen (110 mg twice daily) is broadly comparable to or slightly safer than warfarin for GIB, whereas the 300 mg/day regimen (150 mg twice daily) is associated with increased GI bleeding. Rivaroxaban has also been consistently associated with a higher risk of GIB compared with warfarin. Edoxaban exhibits a clear dose effect: 60 mg once daily increases major GIB, whereas 30 mg once daily reduces major bleeding compared with warfarin.

These comparative signals are supported by a more recent network meta-analysis by Chen (2022), which included 37 randomized controlled trials. At standard doses, apixaban 10 mg/day (5 mg twice daily) was associated with a lower risk of major GIB than rivaroxaban ≥15 mg/day, dabigatran 300 mg/day, and edoxaban 60 mg/day [16]. At reduced doses, no statistically significant differences were observed among apixaban 5 mg/day (2.5 mg twice daily), edoxaban 30 mg/day, and dabigatran 220 mg/day (110 mg twice daily); additionally, edoxaban 30 mg/day was associated with a lower risk of GIB than rivaroxaban 10 mg/day [16].

Overall, evidence from randomized trials and meta-analytic comparisons indicates clinically relevant intra-class heterogeneity among DOACs: apixaban appears to have the most favorable gastrointestinal safety profile, rivaroxaban and higher-dose dabigatran are associated with increased GIB risk, and edoxaban shows a dose-dependent pattern [15,16].

### 3.2. Evidence from Real-World Data

Real-world evidence supports the preferential use of DOACs over vitamin K antagonists and, across multiple observational datasets, consistently suggests that apixaban is associated with the most favorable gastrointestinal safety profile among currently available DOACs. Representative findings from large real-world studies are summarized in Table 1.

Large population-based cohorts and meta-analyses of real-world data have consistently demonstrated clinically relevant intra-class heterogeneity among DOACs, with apixaban exhibiting the lowest risk of gastrointestinal bleeding, rivaroxaban the highest, and dabigatran an intermediate, dose-dependent risk profile [17,18,19,20]. Across diverse healthcare systems and observational designs, apixaban has been associated with significantly lower rates of gastrointestinal bleeding than rivaroxaban and with a similar or lower risk than dabigatran and edoxaban [17,18,19]. These associations appear consistent across age strata and baseline risk categories and are concordant with the comparative patterns observed in randomized controlled trials [15,16].

These differences suggest that anticoagulant selection should not be based solely on thromboembolic risk, but should also incorporate gastrointestinal safety, particularly in high-risk patients.

These findings underscore the importance of integrating gastrointestinal risk into anticoagulant selection and preventive strategy.

## 4. Etiology and Clinical Presentation of Gastrointestinal Bleeding

Dabigatran is a notable exception among DOACs because its tartaric acid excipient has been associated with upper gastrointestinal symptoms (e.g., dyspepsia) and may contribute to superficial mucosal irritation or erosions. By contrast, the other DOACs are not considered to cause direct mucosal injury in the manner of nonsteroidal anti-inflammatory drugs (NSAIDs) such as ibuprofen or diclofenac. Nevertheless, all DOACs can precipitate clinically significant bleeding in the presence of pre-existing-or early, previously unrecognized-gastrointestinal lesions through their systemic anticoagulant effect and, in some circumstances, through a local intraluminal anticoagulant effect. Therefore, careful baseline assessment and ongoing clinical vigilance are essential to identify gastrointestinal lesions with hemorrhagic potential that may be unmasked or exacerbated by anticoagulant therapy [15,16,17].

**Figure 2:**
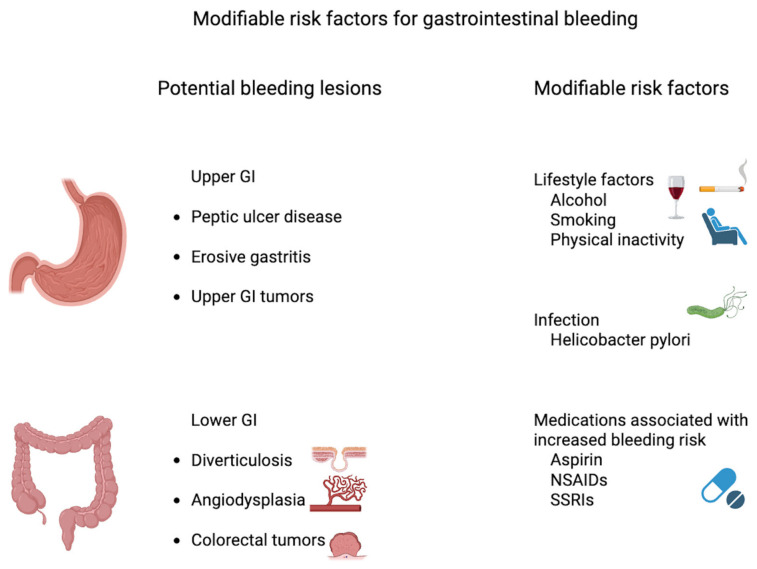
Potential gastrointestinal bleeding sources and modifiable risk factors in DOAC-treated patients. GI, gastrointestinal; NSAIDs, nonsteroidal anti-inflammatory drugs; SSRIs, selective serotonin reuptake inhibitors. Created in BioRender. DUBEI, N. (2026) https://BioRender.com/ti5otg0.

Clinically, gastrointestinal bleeding may be overt-manifesting as hematemesis, melena, or hematochezia-or occult, presenting with symptoms and laboratory features consistent with iron-deficiency anemia. As illustrated in Figure 2, potential upper and lower gastrointestinal bleeding lesions and modifiable bleeding risk factors should be systematically considered in patients receiving direct oral anticoagulants.

### 4.1. Upper Gastrointestinal Bleeding (UGIB)

The most common causes of upper gastrointestinal bleeding (UGIB) are peptic ulcer disease (32–36%) and erosive lesions, including esophagitis (≈24%), gastritis (18–22%), and duodenitis (≈13%) [21]. These conditions are most frequently associated with *Helicobacter pylori* infection and exposure to nonsteroidal anti-inflammatory drugs.

#### 4.1.1. *Helicobacter pylori* Infection

The prevalence of *Helicobacter pylori* (*H. pylori*) infection varies substantially worldwide, ranging from approximately 34–36% in the United States and Western Europe to 69–70% in South America and Africa [22]. *H. pylori* plays a central etiologic role in peptic ulcer disease, being implicated in roughly 95% of duodenal ulcers and 70% of gastric ulcers [23], and it is also strongly associated with gastric malignancy [24]. In patients receiving NSAIDs, concomitant *H. pylori* infection approximately doubles the risk of UGIB, whereas eradication therapy significantly reduces bleeding incidence [25]. Accordingly, the Houston Consensus recommends testing for and eradicating *H. pylori* before initiating long-term NSAID therapy (>4 weeks) [26]. In line with this, the European Maastricht VI/Florence Consensus similarly recommends *H. pylori* testing and eradication prior to long-term NSAID therapy in positive patients. Notably, in patients at high gastrointestinal risk, eradication alone is insufficient, and concomitant PPI therapy remains necessary [27].

Beyond NSAID-related ulceration, *H. pylori* eradication has preventive value in antithrombotic-treated populations. Chan et al. (2013) reported that, among patients receiving chronic aspirin therapy, long-term recurrence of bleeding ulcers was lower in those who underwent *H. pylori* eradication [28], supporting eradication as a risk-reduction strategy, particularly in high-risk individuals.

Real-world data further suggest clinical relevance in DOAC-treated patients. In a retrospective study, Suceveanu et al. found that DOAC recipients with untreated *H. pylori* infection had approximately twice the risk of gastric bleeding compared with those in whom eradication had been achieved; untreated infection was also associated with more severe bleeding, greater need for combined endoscopic hemostatic techniques (e.g., epinephrine injection and hemostatic clip application), and higher mortality. Conversely, eradication was associated with fewer UGIB events, reduced need for surgery, and lower mortality [29].

Population-level evidence is consistent with these observations. Jiang et al. compared hospitalization for UGIB among patients who underwent *H. pylori* eradication prior to initiating oral anticoagulation (warfarin or DOACs) with that of *H. pylori*-negative patients. DOAC-treated patients had a lower risk of UGIB hospitalization than those receiving warfarin (HR 0.26; 95% CI 0.09 to 0.71), and after eradication, UGIB risk in previously infected patients approximated that of individuals without prior infection [30]. Collectively, these findings support screening for and treating *H. pylori* before initiating anticoagulation, particularly in patients with dyspeptic symptoms, prior ulcer disease, anemia, or other UGIB risk features [26,28,29,30].

The Maastricht VI/Florence Consensus (Statement 10) notes that there is currently no evidence that anticoagulants—including DOACs—increase the risk of bleeding in patients with *H. pylori* infection and does not recommend routine screening prior to anticoagulant initiation. Nevertheless, in patients at high gastrointestinal risk, testing for *H. pylori* infection as part of the baseline evaluation (Step 1, Section 5.3) remains clinically prudent, consistent with the broader principle of risk stratification before long-term anticoagulation [27].

Despite strong evidence supporting eradication, the absence of a standard screening strategy remains a relevant gap in clinical practice.

#### 4.1.2. Medications

##### Nonsteroidal Anti-Inflammatory Drugs

NSAID use represents one of the most consistent and clinically relevant drivers of gastrointestinal bleeding in anticoagulated patients, and its avoidance should be considered a cornerstone of preventive strategies. NSAIDs induce direct gastrointestinal mucosal injury and can increase the risk of gastrointestinal bleeding by up to fivefold [31]. Cyclooxygenase-2 (COX-2) selective inhibitors (e.g., celecoxib) are associated with lower rates of peptic ulcer disease and GI bleeding than non-selective NSAIDs; however, this advantage is attenuated when co-administered with aspirin.

NSAID exposure is common in older adults, given the high and increasing global burden of osteoarthritis, particularly in an ageing population [32], and approximately two-thirds of patients with atrial fibrillation are older than 75 years and thus frequently meet indications for anticoagulation [33]. As a result, concomitant NSAID use is a frequent and clinically important driver of bleeding risk in anticoagulated populations.

In a Danish nationwide cohort of 51,794 patients treated with oral anticoagulation for venous thromboembolism and followed for 11 years, concomitant NSAID use was associated with an approximately twofold increase in GI bleeding (adjusted HR 2.24; 95% CI 1.61–3.11), as well as increased intracranial hemorrhage (HR 2.09; 95% CI 1.67–2.62) and bleeding-related anemia (HR 2.99; 95% CI 1.45–6.18) [34]. The risk varied across NSAID molecules, with higher estimates reported for diclofenac and naproxen than for ibuprofen [34]. Among COX-2 inhibitors, celecoxib has been associated with a lower bleeding risk than non-selective NSAIDs when used concomitantly with oral anticoagulants [35].

Preventive strategies are supported by meta-analytic evidence. A systematic review and meta-analysis reported that antisecretory therapy—particularly proton pump inhibitors (PPIs)—was associated with a significant reduction in UGIB among patients receiving oral anticoagulants (RR 0.67; 95% CI 0.61–0.74), with the greatest benefit observed in those with GI risk factors and in patients receiving concomitant NSAIDs or antiplatelet agents [36]. Consistent with this, contemporary EHRA/ESC/AHA guidance emphasizes avoiding NSAIDs during anticoagulation whenever possible and supports PPI co-therapy in high-risk patients [36]. Clinically, safer analgesic alternatives (e.g., paracetamol/acetaminophen, non-NSAID agents, or topical NSAIDs where appropriate) should be prioritized. A newer non-opioid analgesic agent is emerging as potential NSAID-sparing option; suzetrigine, a selective Nav1.8 sodium channel inhibitor approved by the FDA in 2025 for moderate-to-severe acute pain, may represent a future alternative for patients at high gastrointestinal bleeding risk [37].

##### Antiplatelet Agents

Low-dose aspirin and clopidogrel each confer an approximately twofold increase in UGIB risk, while dual antiplatelet therapy (aspirin plus clopidogrel) is associated with an even greater increase (≈3.7-fold) [38]. Given this elevated risk, guidelines recommend concomitant PPI therapy in patients receiving dual antiplatelet therapy, particularly when additional risk factors are present [38].

The Maastricht VI/Florence Consensus further recommends *H. pylori* testing and treatment in high-risk patients on long-term aspirin therapy, with additional PPI therapy indicated in those at elevated gastrointestinal risk [27].

Drug combinations further amplify risk. UGIB risk increases substantially when low-dose aspirin is combined with NSAIDs across dose ranges (RR ≈2.6) or with high-dose oral corticosteroids (RR 4.43) [9]. These combinations should therefore be avoided or tightly time-limited whenever feasible, and gastroprotection should be considered when they are unavoidable [9,36,38].

##### Selective Serotonin Reuptake Inhibitors

Selective serotonin reuptake inhibitors—including citalopram, escitalopram, fluoxetine, sertraline, paroxetine, and fluvoxamine—are widely prescribed in older adults, accounting for approximately 60% of antidepressant prescriptions in this population [39] and represent an under-recognized contributor to gastrointestinal bleeding risk. Proposed mechanisms include impaired platelet aggregation and prolonged bleeding time due to reduced platelet serotonin content [40,41], increased gastric acid secretion [42], and pharmacokinetic interactions mediated through cytochrome P450 inhibition that may increase exposure to co-administered agents associated with bleeding (e.g., NSAIDs and antiplatelets) [43,44].

Observational evidence suggests that SSRI use is associated with an approximately 55% increase in GIB risk, and that concomitant SSRI plus NSAID therapy may increase bleeding risk several-fold [45]. In antiplatelet-treated populations, addition of an SSRI to aspirin has been associated with increased bleeding (HR 1.42), while triple therapy (aspirin, clopidogrel, and an SSRI) confers higher risk (HR 2.35); combining an SSRI with dual antiplatelet therapy further increases bleeding risk (HR 1.57) [44]. Thus, SSRI therapy should be recognized as an independent bleeding risk factor, particularly when combined with antiplatelet agents or anticoagulation. Where clinically appropriate, risk mitigation may include minimizing additional medication with potential gastrointestinal adverse effects and considering concomitant gastroprotection in high-risk individuals [45].

The cumulative effect of multiple medications represents a frequently underestimated contributor to gastrointestinal bleeding risk.

### 4.2. Lower Gastrointestinal Bleeding (LGIB)

The most common etiologies of lower gastrointestinal bleeding (LGIB) include diverticular disease (20–50%), hemorrhoids (20–21%), colorectal polyps (≈13%), colorectal neoplasms (≈8%), angiodysplasia (3–6%), and ischemic colitis (≈6.6%) [46,47,48,49]. In a cohort study by Aoki et al., LGIB was reported more frequently than upper gastrointestinal bleeding, particularly among patients treated with dabigatran; the leading causes, in descending order, were diverticulosis, telangiectasia, and hemorrhoids [50].

#### 4.2.1. Diverticular Disease

Diverticular bleeding predominantly affects older adults, reflecting both the age-related prevalence of diverticulosis and the clustering of vascular comorbidities in this population. Diverticular disease is present in approximately 5% of individuals <40 years and in up to 65% of those >85 years in the United States and Western Europe [51]. Major risk factors for diverticular bleeding include arterial hypertension, diabetes mellitus, and coronary artery disease [52,53,54]. In addition, exposure to NSAIDs, oral anticoagulants, and low-dose aspirin further increases the risk of diverticular hemorrhage [55].

Notably, the epidemiology of LGIB differs substantially across regions. While diverticulosis is the leading cause of LGIB in many Western countries—often linked to low-fiber dietary patterns—it accounts for only 1.1% of cases in China, where the predominant etiologies include colorectal cancer (24.4%), colorectal polyps (24.1%), and chronic colitis (16.8%) [56]. These geographic differences have important implications for risk stratification and diagnostic strategies in anticoagulated patients.

#### 4.2.2. Angiodysplasia

Angiodysplasia is being recognized with increasing frequency, partly due to improved detection through colorectal cancer screening programs and partly because the expanding use of antithrombotic therapy facilitates clinically apparent bleeding from otherwise subclinical lesions. In parallel with population aging and broader antithrombotic use, angiodysplasia-related bleeding episodes appear to be increasing. A key clinical challenge is the high propensity for recurrence. In the study by Khan et al., approximately one in five patients experienced recurrent bleeding within 30 days after hospital discharge, underscoring the need for careful follow-up and secondary prevention strategies in this subgroup [57].

A clinically relevant association exists between severe aortic stenosis and recurrent gastrointestinal bleeding from angiodysplasia, a condition known as Heyde syndrome. The underlying mechanism involves acquired von Willebrand factor deficiency secondary to high shear across the stenotic valve. In patients with this association who are receiving anticoagulant therapy, transcatheter aortic valve implantation (TAVI, also referred to as transcatheter aortic valve replacement [TAVR] in North American literature) may reduce the frequency of recurrent bleeding episodes—with cessation of gastrointestinal bleeding reported in up to 83% of patients at 5 years—and should be considered as part of the preventive strategy in eligible patients [58,59].

#### 4.2.3. Colorectal Polyps and Neoplasia: Anticoagulation May Act as a “Bleeding Stress Test”, Unmasking Previously Undiagnosed Gastrointestinal Pathology

Colorectal polyps and malignancies are frequently identified following bleeding episodes during anticoagulant therapy, which may unmask pre-existing lesions. In a large cohort study by Grewal et al. including 119,480 patients newly initiated on anticoagulants and followed for two years, 21.8% experienced a bleeding event and 4.9% (≈5800 patients) were diagnosed with cancer during follow-up [60]. Bleeding was strongly associated with subsequent cancer diagnosis (HR 4.0 for any cancer and HR 5.0 for gastrointestinal cancers), and tumors detected after a bleeding event were, on average, diagnosed at an earlier stage than those identified without antecedent bleeding [60]. These observations support the concept of anticoagulation as a “bleeding stress test”, reinforcing that any LGIB occurring during anticoagulant therapy warrants a structured diagnostic evaluation to identify the underlying lesion. This principle may be particularly important in settings without established screening programs, where the proportion of bleeding events attributable to benign or malignant neoplasia is likely higher [56,60].

In patients initiating DOAC therapy who are not enrolled in a colorectal cancer screening program, pre-treatment evaluation for colorectal lesions should be considered. A stepwise approach—FIT as initial screening, with colonoscopy in FIT-positive patients—is discussed in detail in Section 5.1. When polypectomy is required in patients already receiving DOACs, anticoagulation management—including timing of DOAC interruption and resumption—should follow current clinical guidelines [14].

### 4.3. Clinical Uncertainties and Controversies

Although the available evidence on gastrointestinal bleeding (GIB) in patients treated with direct oral anticoagulants (DOACs) is increasing, several areas of clinical uncertainty remain insufficiently addressed in current guidelines and existing reviews. These gaps are particularly relevant in real-world decision-making, where individualized risk assessment and competing clinical priorities often complicate management strategies.

A key unresolved issue concerns the role of proton pump inhibitor (PPI) co-therapy. While PPI use is consistently associated with a reduction in upper gastrointestinal bleeding, the optimal strategy for its use in DOAC-treated patients remains unclear. Current recommendations generally support PPI use in high-risk individuals; however, whether routine prophylactic use should be extended to broader patient populations remains controversial. The potential benefits of wider PPI use must be balanced against concerns regarding long-term adverse effects and the risk of overtreatment in low-risk patients.

Similarly, the question of *Helicobacter pylori* screening and eradication in patients initiating DOAC therapy lacks a standardized approach. Although eradication clearly reduces the risk of peptic ulcer-related bleeding, particularly in patients receiving NSAIDs or antiplatelet therapy, there is insufficient evidence to support universal screening prior to anticoagulation. A more selective strategy targeting patients with dyspeptic symptoms, prior ulcer disease, or anemia may be more appropriate, but this approach has not been formally validated in prospective studies.

The clinical relevance of differences in gastrointestinal bleeding risk among individual DOACs also remains a matter of debate. While randomized trials and real-world studies consistently suggest a more favorable gastrointestinal safety profile for apixaban compared with rivaroxaban and higher-dose dabigatran, it remains uncertain to what extent these differences should influence anticoagulant selection in individual patients. In practice, the choice of DOAC is often driven by thromboembolic risk, renal function, and drug interactions, with gastrointestinal risk considered only secondarily. Whether a GI-focused anticoagulant selection strategy improves clinical outcomes has not been definitively established.

Another area of uncertainty relates to the management of lower gastrointestinal bleeding risk, which is less well characterized than upper gastrointestinal bleeding. In contrast to peptic ulcer disease, many lower gastrointestinal lesions-such as angiodysplasia, diverticulosis, or colorectal neoplasia-may remain clinically silent until bleeding occurs. This raises the important question of whether DOAC-associated bleeding should be viewed not only as an adverse event but as a potential marker of underlying gastrointestinal pathology. In this context, anticoagulation may act as a “bleeding stress test”, unmasking previously undiagnosed lesions that warrant targeted investigation.

Taken together, these uncertainties highlight the limitations of a purely descriptive approach to gastrointestinal bleeding risk in DOAC-treated patients. They underscore the need for a structured, clinically oriented framework that supports individualized decision-making based on the interaction between patient-specific factors, gastrointestinal pathology, and treatment-related risks.

**Figure 3:**
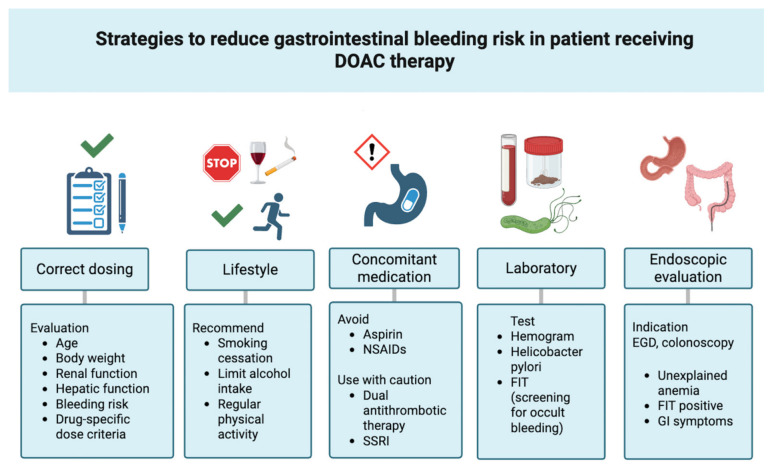
A site-specific framework for prevention of DOAC-associated gastrointestinal bleeding. EGD, esophagogastroduodenoscopy; GI, gastrointestinal; FIT, fecal immunochemical testing; NSAIDs, nonsteroidal anti-inflammatory drugs; SSRIs, selective serotonin reuptake inhibitors. Created in BioRender. DUBEI, N. (2026) https://BioRender.com/vpjfr63.

Based on these considerations, to facilitate translation of the available evidence into everyday clinical practice, we propose a pragmatic tiered management framework based on individual gastrointestinal bleeding risk, with specific preventive and monitoring strategies according to low-, intermediate-, and high-risk profiles (Table 2). This table provides a simplified risk stratification framework, whereas the algorithm presented in Figure 3 translates these categories into a stepwise approach to clinical decision-making.

## 5. Practical Strategies for Prevention of Gastrointestinal Bleeding

The following strategies expand on the interventions summarized in Table 2 and provide additional clinical context to support individualized decision-making in routine practice.

### 5.1. Before Starting DOAC Therapy

In patients with atrial fibrillation, direct oral anticoagulants are preferred over vitamin K antagonists for the prevention of stroke and systemic embolism because they provide a more favorable efficacy–safety profile. Nonetheless, DOAC therapy should be avoided in the presence of specific contraindications.

Appropriate dose selection is a key determinant of both effectiveness and safety. Reduced-dose regimens should be prescribed only to patients who meet validated dose-reduction criteria, with careful consideration of age, body weight, renal function, and relevant concomitant medications (e.g., verapamil with dabigatran; cyclosporine, dronedarone, erythromycin, or ketoconazole with edoxaban). For dabigatran in particular, pre-existing upper gastrointestinal conditions—such as esophagitis, gastro-esophageal reflux disease, or gastritis—should be explicitly assessed, given its recognized association with dyspeptic symptoms [15,16].

Beyond dyspeptic symptoms, these conditions carry pharmacokinetic relevance. Dabigatran etexilate is a prodrug requiring an acidic environment for absorption; however, its capsule formulation contains tartaric acid that creates a local acidic micro-environment, partially mitigating this pH dependence. Although concomitant PPI use reduces dabigatran Cmax at steady state by approximately 28%, this is not considered clinically significant and they can be safely co-administered. The pharmacokinetics of rivaroxaban, apixaban, and edoxaban are not meaningfully affected by PPI co-administration [61]. Nevertheless, the SPC for dabigatran specifically identifies pre-existing upper gastrointestinal conditions as factors associated with increased bleeding risk, independent of any pharmacokinetic interaction. In patients with such conditions, the choice of DOAC should therefore be individualized, and switching to an alternative agent may be considered based on the overall gastrointestinal risk profile.

Systematic identification and management of modifiable bleeding risk factors are essential. These include lifestyle exposures, concomitant medications that increase bleeding risk, and *Helicobacter pylori* infection.

Lifestyle interventions may contribute to lowering gastrointestinal bleeding risk. Smoking cessation and limitation of alcohol intake should be recommended. Regular physical activity should be encouraged and may also have relevance for the prevention of diverticular disease [15].

Patients should receive clear counseling regarding concomitant medications, avoidance of self-medication, and the need to avoid aspirin and NSAIDs. When analgesia is required, safer alternatives should be prioritized (e.g., paracetamol/acetaminophen, non-NSAID analgesics, topical therapies, or emerging non-opioid agents such as suzetrigine). In patients receiving selective serotonin reuptake inhibitors, particularly when combined with antithrombotic therapy, reassessment of antidepressant treatment in collaboration with psychiatry should be considered when clinically appropriate [15]. Among modifiable factors, NSAID use represents one of the most consistent and clinically relevant drivers of gastrointestinal bleeding and should be actively avoided whenever possible.

Testing for *H. pylori*, provision of eradication therapy when positive, and confirmation of successful eradication should be systematically considered in high-risk patients [23,24].

Patients with iron-deficiency anemia should undergo structured evaluation, typically including both upper endoscopy and colonoscopy, to identify occult bleeding sources.

For individuals who are not enrolled in colorectal cancer screening programs, fecal immunochemical testing (FIT) may serve as an initial screening tool; patients with positive results should be referred for colonoscopy [15,16,17].

Finally, prophylactic proton pump inhibitor therapy should be considered in patients at increased risk of upper gastrointestinal bleeding; however, its routine use in low-risk individuals remains controversial and should be individualized based on overall risk profile. Current guidelines reflect a growing trend toward reassessment and de-prescribing of proton pump inhibitors in the general population. However, the AGA Clinical Practice Update on De-Prescribing of Proton Pump Inhibitors [62] explicitly recommends that all PPI users be assessed for upper gastrointestinal bleeding risk before any de-prescribing is considered (Best Practice Advice 6), and that patients at high risk for upper gastrointestinal bleeding should not be considered for PPI discontinuation (Best Practice Advice 7). DOAC-treated patients with concomitant gastrointestinal risk factors represent a high-risk population in whom PPI co-therapy should be maintained [62].

### 5.2. During Follow-Up

Regular follow-up is essential and should include surveillance of body weight and periodic assessment of renal and hepatic function to enable timely dose optimization when indicated.

Bleeding risk should be reassessed at regular intervals using validated scores (e.g., HAS-BLED). Importantly, these tools should not be used to justify withholding or discontinuing anticoagulation when it is otherwise indicated; rather, they should support structured identification and correction of modifiable risk factors over time [12].

Serial complete blood counts should be obtained during follow-up to facilitate early detection of anemia. In patients without prior colorectal cancer screening, fecal immunochemical testing (FIT) performed at DOAC initiation represents a pragmatic first step, as described in Section 5.1. In those with a negative initial FIT result, annual FIT may be continued as part of an ongoing colorectal cancer screening strategy. The rationale is that early detection of newly developing colorectal lesions—polyps or neoplasia—in patients receiving long-term anticoagulation may reduce the risk of future clinically significant gastrointestinal bleeding. It should be noted that FIT in this context serves as a colorectal cancer screening tool and not as a dedicated surveillance strategy for anticoagulation-induced occult bleeding; its role specifically in DOAC-treated population has not been formally validated and reflects clinical judgment. The development of iron-deficiency anemia and/or a positive FIT result should prompt diagnostic endoscopic assessment—typically including both upper endoscopy and colonoscopy—to identify the bleeding source ang guide targeted management [50].

### 5.3. Proposed Risk-Based Clinical Algorithm for Gastrointestinal Bleeding Prevention

In response to the clinical challenges and uncertainties outlined above, we propose a decision-oriented algorithm for the prevention of gastrointestinal bleeding in patients receiving DOAC therapy. This algorithm is intended to guide clinical decision-making in routine practice rather than to replace guideline-based recommendations.

**Step 1: Assess baseline gastrointestinal bleeding risk before DOAC initiation**

Before prescribing a DOAC, clinicians should systematically evaluate the following:

• Prior gastrointestinal bleeding;
• Iron-deficiency anemia or unexplained anemia;
• Dyspepsia, gastroesophageal reflux, or prior peptic ulcer disease;
• Known or suspected gastrointestinal lesions (e.g., angiodysplasia, diverticulosis, colorectal polyps, malignancy);
• Concomitant use of NSAIDs, antiplatelet agents, corticosteroids, or SSRIs;
• Age, body weight, renal function, and potential drug interactions;
• *H. pylori* status in selected high-risk patients;
• Colorectal cancer screening status and need for evaluation of occult bleeding.

**Step 2: Identify modifiable risk factors**

The main modifiable contributors to DOAC-related GIB include:

• Unnecessary NSAID exposure;
• Avoidable concomitant antiplatelet therapy;
• Untreated *H. pylori* infection;
• Off-label DOAC dosing;
• Lack of gastroprotection in patients at high UGIB risk;
• Uninvestigated anemia or occult gastrointestinal bleeding.

**Step 3: Select the most appropriate anticoagulant strategy**

When anticoagulation is indicated, DOAC choice and dose should be individualized according to thromboembolic indication, renal function, label-based dose criteria, and gastrointestinal risk profile. In patients at increased GI bleeding risk, a DOAC with a more favorable GI safety profile, particularly apixaban, may be preferred when clinically appropriate.

**Step 4: Apply targeted preventive interventions**

Depending on the bleeding phenotype and risk profile, preventive strategies may include:

• Avoidance of NSAIDs whenever possible;
• Reassessment of the indication for concomitant antiplatelet therapy;
• Testing for and eradication of *H. pylori* in selected patients;
• Proton pump inhibitor co-therapy in patients at high risk of upper GI bleeding;
• Use of safer analgesic alternatives;
• Endoscopic evaluation in patients with iron-deficiency anemia, prior GI bleeding, positive FIT, or persistent GI symptoms.

**Step 5: Stratify follow-up intensity**

Follow-up should be tailored to individual GI bleeding risk:

• Low risk: routine follow-up, periodic CBC, reinforcement of warning symptoms and avoidance of self-medication;
• Intermediate risk: closer review of symptoms, medication exposure, renal function, and need for gastroprotection;
• High risk: frequent CBC monitoring, low threshold for endoscopic investigation, repeated reassessment of antithrombotic combinations, and maintenance of corrective measures for reversible risk factors.

**Step 6: Reassess risk dynamically during treatment**

Gastrointestinal bleeding risk is not static. It should be reassessed during follow-up, especially after:

• New anemia;
• Dyspepsia or GI symptoms;
• Initiation of NSAIDs, antiplatelets, or SSRIs;
• Decline in renal function;
• Any overt or occult bleeding event.

Figure 3 summarizes a pragmatic, stepwise approach to gastrointestinal bleeding prevention in patients receiving DOAC therapy, highlighting interventions to be implemented at treatment initiation and reinforced during follow-up.

This algorithm integrates baseline risk assessment, stratification, and targeted preventive strategies into a structured and clinically applicable approach. Central to safe DOAC prescribing is correct dosing, which requires systematic evaluation of patient-specific factors known to alter drug exposure and bleeding risk: age, body weight, renal function, and concomitant medications with pharmacokinetic interactions. These factors are not static—renal function may deteriorate during long-term anticoagulation, progressively increasing DOAC plasma concentration and bleeding risk, and should be reassessed at regular intervals as outlined in Steps 5 and 6.

## 6. Limitations and Practical Implications

This narrative review has several limitations that should be acknowledged. By design, narrative synthesis does not follow a fully reproducible search and selection methodology; therefore, the included literature and its interpretation may be susceptible to selection and reporting bias. In addition, the available evidence on gastrointestinal bleeding in patients treated with direct oral anticoagulants is heterogeneous, with substantial variability in baseline patient risk, comorbidity burden, concomitant antithrombotic exposure, definitions of bleeding severity and anatomical site, and duration of follow-up. Finally, for several preventive strategies—particularly lifestyle and nutritional interventions—the strength of evidence is constrained by a relative paucity of adequately powered randomized trials. As a result, recommendations in these domains are informed predominantly by observational studies and expert consensus rather than definitive causal data.

Despite these limitations, this review offers clinically relevant and actionable insights. It integrates mechanistic considerations with data from randomized trials and real-world cohorts. It proposes a structured, pragmatic approach for evaluating and reducing gastrointestinal bleeding risk in patients receiving DOAC therapy, both at treatment initiation and during longitudinal follow-up. By prioritizing the identification and correction of modifiable risk factors (e.g., concomitant medications that increase gastrointestinal bleeding risk, untreated *H. pylori* infection, and occult anemia), reinforcing patient education to minimize avoidable exposures, and outlining indication-driven diagnostic pathways, this framework is readily implementable in routine clinical practice. Collectively, these strategies may support safer anticoagulant prescribing and improve the net clinical benefit of DOAC therapy by helping clinicians maintain effective thromboembolic prevention while minimizing preventable bleeding complications. Importantly, the proposed risk-based framework is not derived from prospective validation but represents a pragmatic synthesis of currently available evidence intended to support clinical decision-making.

## 7. Conclusions

Gastrointestinal bleeding remains the most frequent extracranial complication of DOAC therapy and is associated with adverse outcomes, including treatment interruption and increased mortality. However, gastrointestinal bleeding risk is largely driven by identifiable and often modifiable factors, including underlying gastrointestinal pathology and concomitant medications. The review emphasizes the importance of a site-specific and dynamic approach to bleeding risk assessment, moving beyond global risk scores toward individualized evaluation of gastrointestinal vulnerability.

The systematic identification and correction of modifiable risk factors—such as NSAID use, *Helicobacter pylori* infection, and occult gastrointestinal lesions—represent key opportunities to reduce preventable bleeding events without compromising the benefits of anticoagulation.

A shift toward proactive and individualized gastrointestinal risk assessment, combined with structured follow-up and targeted preventive strategies, is essential to optimize the safety of DOAC therapy while preserving its thromboembolic benefits.

The proposed framework should be regarded as a pragmatic, clinically oriented tool derived from the available evidence rather than a prospectively validated algorithm. Future prospective studies are needed to confirm its clinical utility, impact on patient outcomes, and role in guiding individualized bleeding risk mitigation strategies.

## Figures and Tables

**Table 1:** Representative real-world evidence on gastrointestinal bleeding with DOACs versus VKAs and comparative GIB risk across DOACs in non-valvular atrial fibrillation.

Author/Year	Study Design/Population	Comparisons	Sample Size	GIB Effect Estimate (95% CI)	Key Message	Real-World Relevance
**Cha** **J.M., 2024 [17]**	Meta-analysis of RWE; treatment-naive NVAF	DOAC vs. VKA; comparisons among DOACs	~37 studies; ~2.3 million	Overall DOAC ≈ VKA for GIB; apixaban/edoxaban ↓ GIB vs. rivaroxaban/dabigatran	In routine practice, apixaban shows the most favorable GI profile; consistent intra-class differences across DOACs	Large meta-analysis, but substantial observational heterogeneity
**Yao X., 2016 [18]**	Retrospective U.S. cohort; NVAF initiators of DOAC/VKA	Dabigatran, rivaroxaban, apixaban vs. warfarin	>250,000	Apixaban: ↓ GIB vs. VKA; dabigatran: GIB ≈/↑ vs. VKA; rivaroxaban: ↑ GIB vs. VKA	Apixaban lowest GI risk; rivaroxaban highest; dabigatran intermediate (dose-dependent)	Robust cohort design, but residual confounding remains
**Mamas A.M., 2022 [19]**	Meta-analysis of RWE in NVAF	Apixaban vs. rivaroxaban	>500,000 (10 studies)	Apixaban ↓ GIB vs. rivaroxaban: HR 0.57 (0.50–0.64)	Apixaban demonstrates clearly superior GI safety compared with rivaroxaban	Clinically relevant comparison, limited by observational pooling
**Ingason A.B., 2023 [20]**	Population-based analysis	Warfarin vs. DOAC	~100,000	Warfarin ↑ UGIB vs. DOAC; LGIB similar	DOACs are associated with better UGIB outcomes than warfarin, with comparable LGIB risk	Nationwide analysis, but bleeding-site misclassification remains possible

DOAC, direct oral anticoagulant; VKA, vitamin K antagonist; NVAF, non-valvular atrial fibrillation; GIB, gastrointestinal bleeding; RWE, real-world evidence; ICH, intracranial hemorrhage; HR, hazard ratio; OD, odds ratio; CI, confidence interval; NS, non-significant; UGIB, upper gastrointestinal bleeding; LGIB, lower gastrointestinal bleeding. The arrows indicate a decrease or an increase.

**Table 2:** Proposed tiered management according to gastrointestinal bleeding risk profile in patients receiving DOAC therapy.

GI Risk	Typical Patient Profile	Recommended Preventive Measures	Monitoring During Follow-Up
**Low risk**	Patients without major/minor factors: No prior GIB; no anemia; no known GI lesion; no concomitant NSAID, antiplatelet, or SSRI therapy; no significant upper GI symptoms	Standard DOAC selection according to indication, renal function, and label criteria; avoid unnecessary NSAIDs/aspirin; reinforce lifestyle counseling and warning symptoms	CBC: every 6–12 months; FIT: annually if not included in a colorectal screening program; clinical reassessment at routine visits
**Intermediate risk**	One or more minor GI risk factors: age ≥75 years, dyspepsia/reflux, intermittent NSAID/antiplatelet exposure, SSRI use, prior uncomplicated ulcer disease, suspected diverticular disease	Prefer DOAC with more favorable GI safety profile when clinically appropriate; review interacting drugs; consider *H. pylori* testing/eradication in selected patients; consider PPI if upper GI risk predominates	CBC: every 3–6 months; FIT: annually; reassess GI symptoms, medication exposure, renal function, and need for gastroprotection
**High risk**	One major GI risk factors: Prior major GIB; iron-deficiency anemia; active or recent peptic ulcer disease; untreated *H. pylori* infection; known angiodysplasia, diverticulosis with prior bleeding, colorectal neoplasia, or need for combined antithrombotic therapy	Correct modifiable risk factors before or shortly after DOAC initiation; test and eradicate *H. pylori*; use PPI when upper GI risk is present; avoid NSAIDs whenever possible; minimize concomitant antiplatelet therapy; consider targeted endoscopic evaluation and DOAC selection with lowest GI risk profile when feasible	CBC: every 1–3 months initially, then every 3–6 months once stable; FIT: annually if endoscopic evaluation is not already indicated; low threshold for EGD/colonoscopy in case of anemia, positive FIT, or new GI symptoms

CBC, complete blood count; DOAC, direct oral anticoagulant; EGD, esophagogastroduodenoscopy; FIT, fecal immunochemical test; GI, gastrointestinal; GIB, gastrointestinal bleeding; PPI, proton pump inhibitor; SSRI, selective serotonin reuptake inhibitor.

## Data Availability

No new data were generated or analyzed in this study.

## Abbreviations

The following abbreviations are used in this manuscript:

AF	Atrial fibrillation
DOAC	Direct oral anticoagulant
VKA	Vitamin K antagonist
GIB	Gastrointestinal bleeding
NSAID	Nonsteroidal anti-inflammatory drug
SSRI	Selective serotonin reuptake inhibitor
ESC	European Society of Cardiology
UGIB	Upper Gastrointestinal Bleeding
COX-2	Cyclooxygenase-2
PPI	Proton pump inhibitor
LGIB	Lower Gastrointestinal Bleeding
FIT	Fecal immunochemical testing
EGD	Esophagogastroduodenoscopy
